# A global campaign to combat ageism

**DOI:** 10.2471/BLT.17.202424

**Published:** 2018-03-09

**Authors:** Alana Officer, Vânia de la Fuente-Núñez

**Affiliations:** a Department of Ageing and Life Course, World Health Organization, 20 Avenue Appia, 1211 Geneva 27, Switzerland.; Correspondence to Alana Officer (email: officera@who.int).

The World Health Organization (WHO) defines ageism as the stereotyping, prejudice and discrimination towards people on the basis of age.^1^ Ageism cuts across the life-course and stems from the perception that a person might be too old or too young to be or to do something.

Ageism is highly prevalent;^2^^,^^3^ however, unlike other forms of discrimination, including sexism and racism, it is socially accepted and usually unchallenged, because of its largely implicit and subconscious nature.^4^^,^^5^

Children as young as 4 years are aware of their cultures’ age stereotypes.^6^ These stereotypes focus predominantly on the negative aspects of ageing, with older age typecast as an inevitable decline in physical and mental capacities and a period of dependency. Language and media, including films, television, popular music, print and social media, most often echo and reinforce these stereotypes, because ageist depictions tend to be the norm.^7^^–^^9^ As we get older, we experience ageism from others, but also from ourselves, because of the unconscious internalization of society’s negative attitudes and stereotypes towards older people. This helps to explain why older people often try to stay young, feel shame about getting older and limit what they think they can do instead of taking pride in the accomplishment of ageing.

Perceived discrimination, whether based on race, gender or age, has negative health outcomes.^10^ Ageism has been shown to have significant impact on our participation in society, health and longevity. For example, evidence shows that those who hold negative attitudes on ageing have slower recovery from disability,^11^ live on average 7.5 years less than those who hold positive attitudes^12^ and are less likely to be socially integrated.^13^ Ageism also imposes barriers to the development of good policies on ageing and health as it influences the way problems are framed, the questions that are asked and the solutions that are offered. In this context, age is often understood as sufficient justification for treating people unequally and limiting their opportunities for meaningful contribution.

Experience with sexism and racism has shown that changing social norms is possible and can result in more prosperous and equitable societies. Changing people’s understanding, social behaviours and political determination around age and ageing is possible and essential to foster healthy ageing, the ability for all people to live long and healthy lives and do what they have reason to value.

## A global campaign to combat ageism

Collective, concerted and coordinated global action is required to tackle ageism. Given the current demographic transition, with populations around the world ageing rapidly, we need to act now to generate a positive effect on individuals and society. In May 2016, the 194 WHO Member States called on the organization’s Director-General to develop, in cooperation with other partners, a global campaign to combat ageism.^14^ To be effective, the global campaign to combat ageism must tackle individual and social attitudes, stereotypes and behaviours towards people on the basis of their age, as well as the laws, policies and institutions that either perpetuate ageism or do little to stop it.

To develop the campaign, WHO will build an evidence base on ageism and draw from evidence of what has worked for other public health campaigns, such as end violence against women^15^ and adopt healthier behaviours.^16^ Both campaigns have increased awareness, helped to rally public support and influenced change in individual behaviours and in international and national legislative and policy frameworks.^17^ Evidence suggests that certain conditions need to be met for a campaign to work. In addition to having clear goals and vision, a campaign needs to be evidence-based to understand the nature of the problem, who is affected and how, and which actions should be taken for which target audiences.^18^ The campaign’s approach should include actions that help to change attitudes and behaviours and to develop supportive policy and legal frameworks.^19^ A successful campaign should also be underpinned by a theory of change to anticipate possible routes towards change among target audiences, devise effective implementation strategies^20^ and facilitate evaluation.^17^ The campaign should be multisectoral and multilevel, as well as supportive of monitoring and evaluation. Finally, to ensure sustained action, the campaign should be supported through long-term funding.^21^

Ageism has received little attention in research and policy-making^4^^,^^5^ and the evidence base for global action is yet to be established. There is no global analysis on the magnitude of ageism, its determinants, consequences and what strategies and messages could work to address ageism. To develop the global campaign to combat ageism, WHO needs to find answers to six fundamental questions: (i) what is the global prevalence of ageism? (ii) what are the causes or determinants of ageism? (iii) what are the consequences of ageism at an individual and at a societal level? (iv) what strategies exist to effectively tackle ageism? (v) what are the available metrics to measure the different dimensions of ageism and its implicit and explicit expressions? (vi) What are the most effective ways of building public understanding and expanding thinking about age and ageing?

To start answering these questions, in July 2017 WHO held a meeting with researchers from several universities to outline the methods for conducting a global set of systematic reviews on ageism. The evidence generated from those reviews will help to identify those strategies that are most likely to reduce ageism as well as those populations that should be targeted, either because they affect ageism or because they are affected by it. These reviews will support the development of a tool to measure ageism globally and help to identify key research gaps. As well, the reviews will inform the development of a multicountry study to better understand specific country contexts and ways of communicating around age and ageing. Together these efforts will contribute to identifying a set of core messages that can help shift public understanding, achieving a more age-integrated society.

## Building the foundations for change

To meet the conditions to effectively steer a campaign, in May 2017 WHO convened a meeting of key experts and stakeholders to clarify the campaign’s vision, a world for all ages, and the campaign goal, change the way we think, feel and act towards age and ageing. These, along with the campaign’s principles, have been extensively tested with key stakeholder groups in several languages to ensure global relevance. To support the implementation of the campaign, a draft theory of change was also developed and will be refined based on the findings from the systematic reviews.

Social change requires sustained and coordinated action by a diverse range of public and private sector actors. Beyond the core group, a broader coalition will be established to build the critical mass that is needed to achieve positive changes in the way we think, feel and act towards age and ageing. Key actions will include supporting the dissemination of campaign messaging, implementing specific strategies and monitoring and evaluating of the campaign.

The global campaign to combat ageism will provide a platform to change attitudes towards age and ageing and to work together to build a world inclusive of all ages. These changes are essential to ensure health and wellbeing across the life course and will only be possible through concerted, evidence-based action.
